# Endosomal gene expression: a new indicator for prostate cancer patient prognosis?

**DOI:** 10.18632/oncotarget.6114

**Published:** 2015-10-14

**Authors:** Ian R.D. Johnson, Emma J. Parkinson-Lawrence, Helen Keegan, Cathy D. Spillane, Jacqui Barry-O'Crowley, William R. Watson, Stavros Selemidis, Lisa M. Butler, John J. O'Leary, Doug A. Brooks

**Affiliations:** ^1^ Mechanisms in Cell Biology and Disease Research Group, School of Pharmacy and Medical Sciences, Sansom Institute for Health Research, University of South Australia, Adelaide, SA, Australia; ^2^ Department of Pathology, Coombe Women and Infants University Hospital, Dublin, Ireland; ^3^ Department of Histopathology, Trinity College Dublin, Dublin, Ireland; ^4^ UCD School of Medicine and Medical Science, Conway Institute of Biomolecular and Biomedical Research, University College Dublin, Belfield, Dublin, Ireland; ^5^ Infection and Immunity Program, Biomedicine Discovery Institute, Department of Pharmacology, Monash University, Clayton, VIC, Australia; ^6^ Prostate Cancer Research Group, School of Medicine and Freemasons Centre for Men's Health, University of Adelaide, Adelaide, SA, Australia

**Keywords:** prostate cancer, biomarkers, prognosis, endosomal gene expression, mRNA

## Abstract

Prostate cancer continues to be a major cause of morbidity and mortality in men, but a method for accurate prognosis in these patients is yet to be developed. The recent discovery of altered endosomal biogenesis in prostate cancer has identified a fundamental change in the cell biology of this cancer, which holds great promise for the identification of novel biomarkers that can predict disease outcomes. Here we have identified significantly altered expression of endosomal genes in prostate cancer compared to non-malignant tissue in mRNA microarrays and confirmed these findings by qRT-PCR on fresh-frozen tissue. Importantly, we identified endosomal gene expression patterns that were predictive of patient outcomes. Two endosomal tri-gene signatures were identified from a previously published microarray cohort and had a significant capacity to stratify patient outcomes. The expression of *APPL1*, *RAB5A*, *EEA1*, *PDCD6IP*, *NOX4* and *SORT1* were altered in malignant patient tissue, when compared to indolent and normal prostate tissue. These findings support the initiation of a case-control study using larger cohorts of prostate tissue, with documented patient outcomes, to determine if different combinations of these new biomarkers can accurately predict disease status and clinical progression in prostate cancer patients.

## INTRODUCTION

Prostate cancer is the second most commonly diagnosed cancer in males [1], and the incidence of this disease is predicted to double globally by 2030 (WCRF prostate cancer statistics; http://globocan.iarc.fr, accessed May 2014). More than 1.1 million new cases of prostate cancer are diagnosed each year and two thirds of these patients are from the Western world. The marked increase in the age adjusted incidence rate for prostate cancer has been partly attributed to the prostate specific antigen (PSA) test identifying men without clinical symptoms of the disease. Unfortunately, the PSA biomarker neither discriminates between patients who are at a higher risk of progressive disease/mortality and those who have a more favorable prognosis, nor can it adequately distinguish between prostate cancer and benign pathologies [2, 3]. Therefore, there is a significant need for an effective method to accurately define the prognosis for prostate cancer patients.

The investigation of biomarker expression by microarray analysis in patient cohorts, in relation to known clinical parameters, can be used to develop methods for determining patient prognosis [4-6]. Consequently, gene expression profiles that compare prostate cancer to benign prostatic hyperplasia (BPH), prostatic intraepithelial neoplasia (PIN) and normal prostate tissue have been generated from microarray data [4]. This approach has been utilized to identify the enzyme α-methylacyl-CoA racemase (*AMACR*), which was highly expressed in prostate cancer and may have value as a prognostic marker for the disease [7]. However, prostate cancers display substantial inter- and intra-tumor heterogeneity and the altered expression of a single gene may not be predictive for the wider prostate cancer cell population. In addition, altered gene expression may reflect de-differentiation and progression of tumor growth; for example, *AMACR* is an androgen-regulated gene and exhibits variable expression upon androgen-deprivation therapies or androgen-independent disease progression [8]. Therefore, signatures incorporating multiple genes may be required to improve the accuracy of prostate cancer prognosis.

Commercial tests have recently been developed in an attempt to distinguish between aggressive prostate cancer and indolent disease (reviewed by Sartori & Chan 2014 [9]). The Prolaris^®^ test measures the expression of 46 genes involved in cell cycle progression [10], whilst the Oncotype DX^®^ Prostate Cancer Test measures the expression of genes involved in stromal response, cellular organization, proliferation, basal epithelial function, androgen signaling and stress response [11]. While these tests have entered clinical practice in the USA, and alongside the current PSA blood test, can be used to aid in clinical decision making, they do not predict progression to castrate-resistant cancer or determine responses of cancer cells to therapy [12]. Prostate cancer mRNA microarrays were used in the development of these biomarkers, suggesting that this approach has the potential to identify clinically-relevant new prostate cancer biomarkers.

We recently reported that the biology of endosomes is markedly altered in prostate cancer cells [13, 14] and postulated that the expression of these genes might be predictive of disease progression in prostate cancer patients. Endosomes are essential organelles that are involved in cellular energy metabolism, cell division, intracellular signaling and degradation; and are known to have a role in cancer pathogenesis [15]. For example, endosomal cathepsins have previously been reported to be involved in the process of metastasis [16], presumably through their role in the degradation of extracellular matrix. The endosome system also has a specific capacity to respond to cellular and environmental change and may be altered as the cancer grows. We therefore hypothesized that endosome-related genes will be altered in prostate cancer and provide novel gene biomarkers for use in prostate cancer prognosis.

Here, we have investigated endosomal gene expression in multiple independent prostate cancer cohorts and developed two endosomal gene signatures that were predictive of patient outcome. We have also evaluated endosomal gene expression in fresh-frozen tissue sections from radical prostatectomies and demonstrated a capacity to distinguish indolent from aggressive tumors. This study provides evidence that endosomal genes can distinguish prostate cancer patient outcomes and predict disease progression, warranting further investigation of these findings in larger case-control studies.

## RESULTS

### Altered endosome associated gene expression in the Tomlins microarray patient cohort

**Figure 1:**
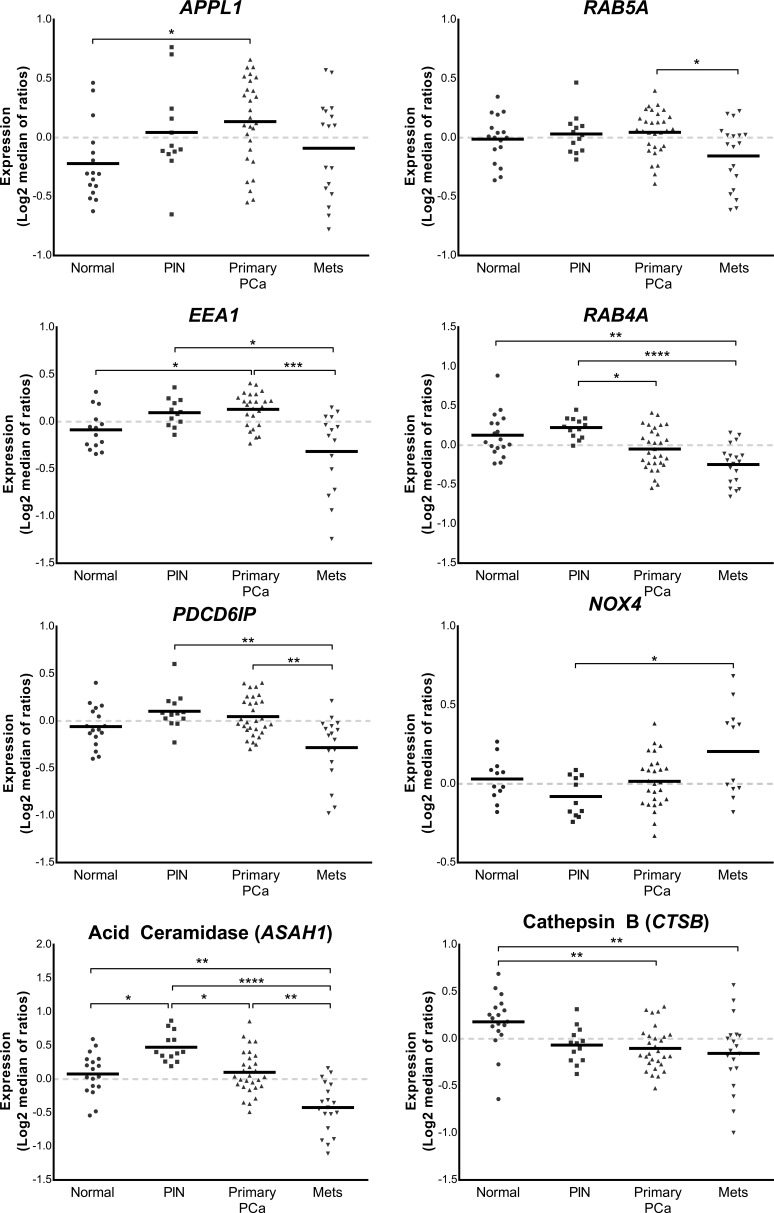
Vertical scatter plots of endosome-associated gene expression data from the Tomlins cohort [17] Expression profiling data derived from the Chinnaiyan Human 20K Hs6 array of 18 non-malignant tissues, 13 prostatic intraepithelial neoplasias, 30 primary prostate cancer and 19 metastatic cancer tissue samples were quantitated to show relative amount of expression of lysosomal-related genes. Statistical significance is represented by an asterisk (*P ≤ 0.05; **P ≤ 0.01; ***P ≤ 0.001; ****P ≤ 0.0001).

The expression of *APPL1* and *EEA1* was significantly increased in primary prostate cancer when compared to non-malignant controls (*P* ≤ 0.05; Figure 1). The expression of *RAB5A* and *EEA1* was significantly reduced in metastatic prostate cancer when compared to primary prostate cancer tissue (*P* ≤ 0.05; Figure 1). The expression of *RAB4A* was significantly decreased in primary prostate cancer when compared to PIN tissue (*P* ≤ 0.05; Figure 1) and there was a significant reduction of *RAB4A* expression in metastatic prostate tissue when compared to both non-malignant prostate cancer (*P* ≤ 0.01) and PIN tissue (*P* ≤ 0.0001; Figure 1). *PDCD6IP* was significantly decreased in metastatic prostate tissue when compared with both primary cancer and PIN tissue (*P* ≤ 0.01). The expression of *NOX4* was significantly increased in metastatic prostate tissue when compared with PIN (*P* ≤ 0.05). Acid ceramidase (*ASAH1)* expression was significantly increased in PIN when compared to non-malignant and primary prostate cancer tissue (*P* ≤ 0.05), and was significantly reduced in metastatic tissue when compared to primary prostate cancer (*P* ≤ 0.01), PIN (*P* ≤ 0.0001), and non-malignant tissue (*P* ≤ 0.01). Cathepsin B (*CTSB*) expression was significantly reduced in both primary cancer tissue and metastatic cancer tissue when compared to non-malignant prostate tissue (*P* ≤ 0.01; Figure 1).

### Endosome associated gene expression is associated with survival outcome in prostate cancer patients

**Figure 2:**
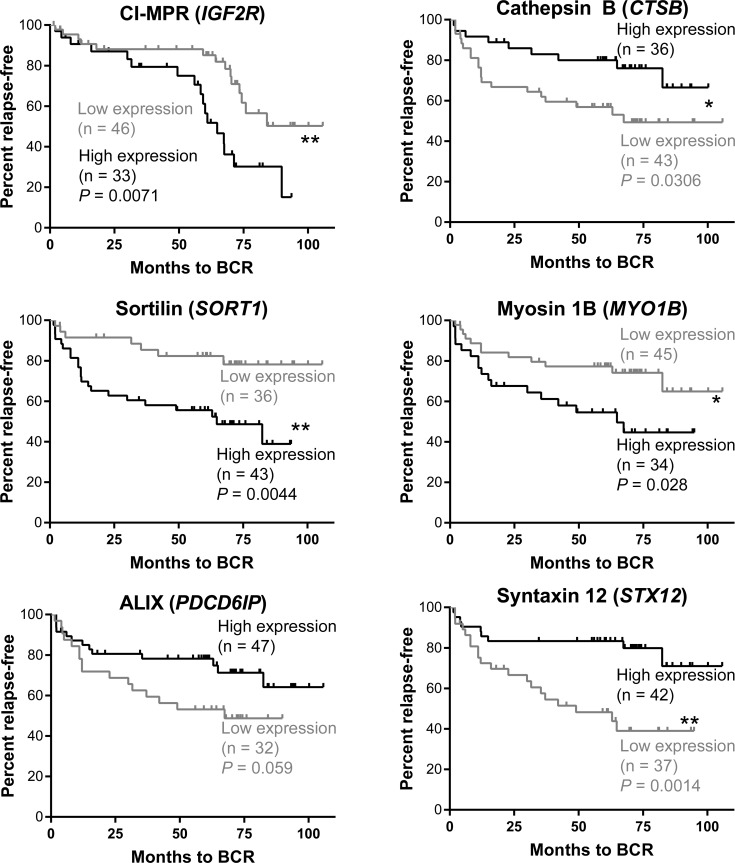
Kaplan-Meier analysis of endosomal genes and patient stratification based on biochemical recurrence (BCR) Patients from the Glinsky cohort [18] were stratified into two groups by *K-*means clustering based on amount (high - black line, low - grey line) of gene expression in prostate cancer samples. Analysis was performed using the Log-Rank test. *IGF2R* (*P* = 0.007), *CTSB* (*P* = 0.03), *MYO1B* (*P* = 0.03), *SORT1* (*P* = 0.004) and *STX12* (*P* = 0.001) differentiated patients at risk of relapse based on the amount of gene expression showed evidence of prognostic capacity.

From the Glinsky cohort [18], patients were classified into two groups of relative high and low mRNA expression of endosome-related genes with an arbitrary cut-point between the two groups defined by *K*-means clustering. There was increased expression of the cation-independent mannose 6-phosphate receptor (*IGF2R*) in patients who had a greater risk of relapse (*P* = 0.007; Figure 2). Clustering of high or low cathepsin B (*CTSB*) expression revealed patients with lower CTSB expression had significantly increased risk of biochemical recurrence (*P* = 0.0306). There was also a significant stratification of patients with Sortilin (*SORT1*) expression, with those patients who expressed greater amounts of *SORT1* at an increased risk of relapse (*P* = 0.004). Myosin 1B (*MYO1B*) stratified patients at risk of recurrence (*P* = 0.03), with patients having increased expression being associated with a poorer prognosis. There was a trend for lower expression of ALIX (*PDCD6IP*) to stratify patients with increased prostate cancer recurrence (*P* = 0.059), and reduced expression of Syntaxin 12 (*STX12*) was also indicative of at-risk patients, with significant stratification (*P* = 0.001; Figure 2).

### Endosomal gene expression can predict clinical outcomes in prostate cancer patients with low amounts of PSA

Analysis from the Tomlins cohort suggested that the mRNA expression of endosome-related genes was altered during disease progression, and that they might therefore have prognostic capacity. Of the patients in the Glinsky cohort that had pre-prostatectomy PSA levels of less than 10 ng/mL, 36.5% had biochemical failure at 100 months (data not shown). We postulated that the altered endosome gene expression in this patient group may indicate changes in cell biology that promote a more aggressive disease, and that this change could stratify patients at risk of recurrence, where the expression of PSA was low or borderline in blood samples.

**Figure 3:**
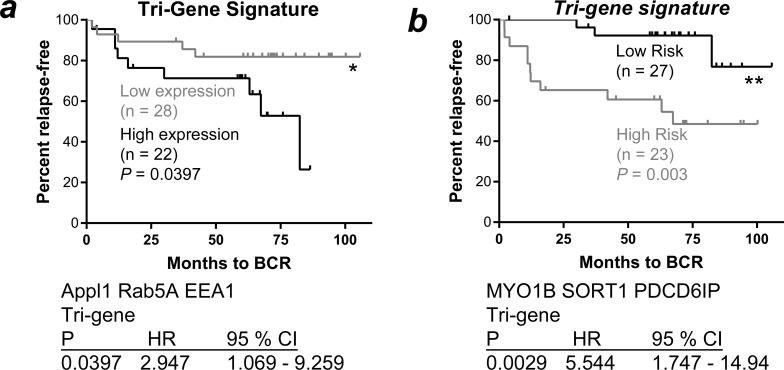
**A.** Kaplan-Meier survival analysis of a combined APPL1, RAB5A and EEA1 gene signature for cancer patients expressing ≤ 10 ng/mL PSA. Patients from the Glinsky cohort [18] expressing PSA ≤ 10 mg/mL were stratified into groups by *K*-means clustering based on *RAB5A*, *APPL1* and *EEA1* gene expression; the three-gene combined signature of *APPL1*, *RAB5A* and *EEA1* stratified patients based on BCR (*P* ≤ 0.0397, Log-Rank test; high expression - black line, low expression - grey line). **B.** Kaplan-Meier survival analysis of *MYO1B*, *PDCD6IP* and *STX12* expression and combined gene signature for cancer patients expressing ≤ 10 ng/mL PSA; the three-gene combined signature of *MYO1B*, *PDCD6IP* and *STX12* stratified patients based on BCR (*P* ≤ 0.0029, Log-Rank test; low risk - black line, high risk - grey line). BCR: biochemical recurrence; HR: hazard ratio; CI: confidence interval.

Combinations of endosomal genes were analyzed to determine their potential for risk stratification, with a focus on genes related to functional endosome machinery in specific spatiotemporal compartments. Stratification of patients based on the expression of a *RAB5A*, *APPL1* and *EEA1* tri-gene signature, using *K*-means clustering methodology, robustly separated patients into two groups with low or high expression of each of the three genes (Figure 3A). Kaplan-Meier survival analysis indicated that patients in the high expression group for this three-gene signature were at significantly higher risk of biochemical recurrence when compared to those in the lower-expression group (HR 2.947, *P* = 0.0397, 95% CI 1.069 - 9.259; Figure 3A). Importantly, stratification of patients based on the expression of a combined MYO1B, PDCD6IP and STX12 tri-gene signature, using K means clustering, robustly separated patients into a low- and high-risk group that showed a greater stratification capacity than any of the single genes (*P* = 0.003; HR 2.947, *P* = 0.0397, 95% CI 1.069 - 9.259; Figure 3B). The high-risk group displayed lower expression of *MYO1B* and increased expression of both *PDCD6IP* and *STX12*.

### qRT-PCR analysis of fresh-frozen prostate tissue revealed significantly altered endosome-associated gene expression in aggressive prostate cancer

**Figure 4:**
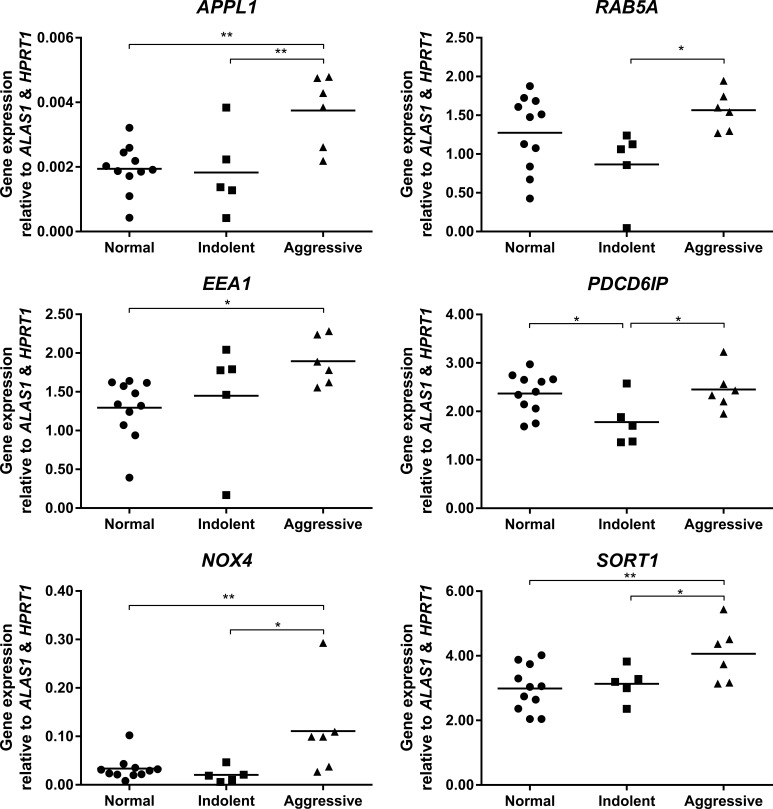
Vertical scatter plots of endosome-associated gene expression from qPCR mRNA analysis on non-malignant (*n* = 11), indolent (*n* = 5) and aggressive (*n* = 6) prostate cancer tissues from the Irish Prostate Cancer Research Consortium, Dublin, Ireland Statistical significance is represented by an asterisk (*P ≤ 0.05; **P ≤ 0.01).

qPCR analysis of endosome associated mRNA in fresh-frozen prostate cancer tissue demonstrated significantly increased expression of *APPL1* in tissue from aggressive prostate cancer compared to non-malignant prostate and indolent prostate cancer tissue (*P* ≤ 0.01; Figure 4). The expression of *RAB5A* and *EEA1* were significantly increased in aggressive cancer tissue compared to indolent diseased tissue (*P* ≤ 0.05). The expression of *NOX4* and *SORT1* were also significantly increased in aggressive cancer tissue when compared with non-malignant (*P* ≤ 0.01) and indolent cancer tissue (*P* ≤ 0.05). There was a significant reduction of *PDCD6IP* mRNA in indolent cancer tissue when compared to both non-malignant and aggressive prostate cancer tissue (*P* ≤ 0.05 respectively).

## DISCUSSION

Prostate cancer is one of the most frequently diagnosed cancers in men, and a leading cause of cancer related deaths worldwide, particularly in the Western world [20, 21]. The PSA test is currently the gold-standard for prostate cancer detection, but is limited for specificity and has limited capacity for prognostic prediction [22]; consequently it cannot readily discriminate patients at higher risk of progressive disease or mortality from those who have a more favorable prognosis. Serum PSA results require contextual interpretation and judgment for each patient, however there are significant problems with specificity. The clinical decision-making process might be improved by the incorporation of multiple prostate cancer biomarkers, especially those that are associated with altered cancer cell biology.

Prognostic nomograms that incorporate continuous as well as categorical variables can potentially be improved by the integration of gene expression analysis and prognostic molecular signatures [23]. Our recent discovery of altered endosome biogenesis in prostate cancer [14] suggests that the biologically relevant changes associated with these endosomal genes have the potential to improve the clinical decision-making process. There are an increasing number of commercial tests for biomarkers that complement the use of PSA (reviewed by Sartori & Chan 2014 [9, 24]), but these tests still do not adequately provide an early accurate prognosis for prostate cancer; and coincidentally, have been discovered anecdotally or through biomarker screening rather than by association with a cell biological process. Gene expression may also be altered throughout the course of the cancer growth and differentiation; for example, α methylacyl CoA racemase is an androgen-regulated gene and exhibits variable expression upon androgen-deprivation therapies or androgen-independent disease progression [8], requiring continued monitoring of these changes. The combined ratio of *PCA3* and *PCA* expression in prostate cells extracted from urine, can indicate the likelihood of prostate cancer, even if biopsies return a negative result [25]; providing a rationale for the use of multiple biomarkers. The altered gene expression in these adjunct tests can elucidate changes in the biology of prostate cancer, such as *PCA3*, which is associated with prostate cancer-cell survival and androgen receptor signaling [26]. This suggests that altered gene expression might need to be linked to functional cell biology to provide new avenues for the development of effective prognostic methodology. However, these new candidate genes can still be investigated in microarray cohorts to help retrospectively determine whether the biomarkers are informative of clinical outcome.

We recently reported that the biogenesis of endosomes is altered in prostate cancer, providing significant data on gene and protein expression in cultured prostate cells [13, 14]. We found that the molecular machinery involved in early endosome biogenesis had altered expression in prostate cancer cells, and that the intracellular location of these compartments was altered, leading to dysregulation of proliferative signals. To establish that these novel cellular changes were clinically relevant, we confirmed here that the endosomal mRNA expression was altered in prostate cancer tissues and was indicative of disease progression; suggesting that these biomarkers might be suitable for prognosis in prostate cancer patients. Our novel gene signatures stratified patients into high and low-risk disease recurrence groups and demonstrated prognostic potential in those patients expressing low or borderline amounts of PSA.

The genes employed for these prognostic signatures are associated with separate populations of early-endosome compartments, thus reflecting that the early endosome population as a whole is disturbed in prostate cancer, rather than one specific compartment, complementing the prior discovery of altered endosome biogenesis in prostate cancer [14]. Altered endosomal-lysosomal biology and the associated changes in gene expression that occur during cancer development may be valuable prognostic indicators; for example, in breast cancer the increased expression of the endosomal protein acid ceramidase (*ASAH1*) has been associated with improved outcomes [27]. Interestingly, *ASAH1* maps to chromosome 8p22, which is frequently deleted in prostate cancer patients [28, 29]. Endosomal *CTSB* (cathepsin B) is also found on chromosome 8p22 and the down regulation of *CTSB* in metastatic tissue may also relate to these chromosomal break points. The reductions in both *ASAH1* and *CTSB* expression in metastatic tissue may therefore be related to this deletion and furthermore is linked to the altered biogenesis of endosomes as these organelles are used to transport both of the latter enzymes.

The changes in *APPL1* (3p21), *RAB5A* (3p24) and *EEA1* (12q22) and *RAB4A* (1q42) in PIN or primary cancer tissue compared to their expression in non-malignant tissue may discriminate metastatic tissue and provide an avenue for monitoring disease progression. The changes in the expression of endosomal genes observed in the Tomlins cohort suggested that they may be involved in the initiation or promotion of pre-malignant cell survival, and that sustained expression is not essential for the continued function of the malignant cell. Indeed, the transgenic adenocarcinoma of the mouse prostate (TRAMP) model of prostate cancer displays elevated gene expression in PIN tissue, which is later reduced in tumors [30]. The wide variation of gene expression changes in the metastatic tissue of the Tomlins cohort, when compared with primary cancer or PIN tissue may reflect the degree of dysfunction in these cells, which in turn contributed to the metastatic cascade. Further study with matched normal and disease samples would ascertain the extent of expression changes for each gene, for the development of a more refined method to predict disease progression.

Microarrays provide only a ‘snapshot’ of a cell population and there is potential for mRNA to be secreted prior to, or post protein-synthesis [31-33], complicating the interpretation of gene expression data. This regulation of the functional gene is equally critical to cancer cell biology and in the case of cathepsin B, the promotion of cancer metastasis through the hydrolytic activity of this enzyme [16]. Thus the incorporation of protein biomarkers and or functional enzymes, analyzed independently of their respective gene expression, may offer further insight into specific cell biology changes and provide more accurate prognosis.

We have noted the limitations of microarray gene expression analysis and consequently performed qPCR analysis of fresh-frozen prostate tissue to confirm our findings on endosomal gene expression. It should be recognized that the Glinsky cohort does not provide information on the gene expression in normal tissue from the cancer patients, or healthy individuals that have increased PSA independent of cancer occurrence, thus the current gene signatures are indicative of prognostic potential rather than necessarily stratifying healthy individuals from those with prostate cancer. Furthermore, incorporating the Gleason scores from the Tomlins and Glinsky cohorts have been precluded from this study, due to the variability in the prognosis from this scoring system [34]. However, a dedicated case-control study utilizing these existing biomarkers and pathology tests, together with the endosomal biomarkers identified here, may result in an improved prognostic assay for prostate cancer.

The significant changes to *APPL1*, *RAB5A* and *EEA1* gene expression in prostate tissue from aggressive disease compared to normal tissue or indolent-disease tissue indicates that these biomarkers have significant potential to monitor disease progression and to stratifying patients at risk of progressive and or metastatic disease. They also have important biological relevance that could provide a therapeutic target, since alterations in endosome biogenesis can result in significantly altered intracellular cell signaling, result in increased cell proliferation and in combination with *NOX4* the expression promote inflammation and angiogenesis [35].

In summary, endosomal gene expression and specific gene signatures have the potential to prediction disease progression in prostate cancer patients and this may lead to improved treatment outcomes. Multivariate risk analysis and stratification of patients into low- and high-risk groups may provide a more individualized approach to prostate cancer patient management, reducing the amount of over-treatment [36]. Active-surveillance is often used for patients deemed to have low-risk prostate cancer (e.g. clinical category T1c, Gleason score ≤ 6, and PSA ≤ 10 ng/mL), but older men are at an increased risk of mortality from prostate cancer, despite low pathology scores and low PSA serum concentrations [37]. Examination of the survival rates for prostate cancer patients expressing low PSA, which is typical of an active surveillance group, reveals some prostate cancers that are aggressive and that result in more rapid recurrence. In the current study, quantitation of gene signatures for the early endosomal genes *APPL1*, *EEA1* and *RAB5A*, and analysis of the endosomal genes *MYO1B*, *PDCD6IP* and *STX12* in prostate cancer patients expressing PSA ≤ 10 ng/mL, led to patient stratification into high and low-risk recurrence groups. These findings justify further clinical investigation of these biomarkers and gene signatures in a dedicated case-control study. Recognizing that prostate cancer patients have altered endosomal gene expression is a significant step towards the development of new, biology-driven biomarkers that could provide accurate prognosis for this important disease.

## MATERIALS AND METHODS

### Patient cohorts

The Tomlins [17] cohort was chosen for its curation of multiple disease stages of prostate cancer. Analysis of tissue samples from this cohort was previously performed using the Chinnaiyan Human 20K Hs6 array [17] and was retrieved from NCBI GEO (accession number GSE6099). It is comprised of 18 non-malignant tissues, 13 prostatic intraepithelial neoplasia's (PIN), 30 primary prostate cancers obtained from radical prostatectomies and 19 metastatic cancer tissue samples were obtained from hormone refractory metastases in the liver, lung or lymph tissue [17]. The Glinsky cohort [18] was obtained from patients who had been treated by radical prostatectomy at Memorial Sloan-Kettering Cancer Center (MSKCC) and was composed of 79 malignant prostate tissues, with clinical follow-up data over 8 years, which was used to assess prostate cancer biochemical recurrence. This cohort comprised 29 patients with biochemical recurrence as defined by a PSA concentration ≥ 0.2 ng/mL and 50 patients with no disease progression. Samples in this cohort had been examined histologically using H&E-stained cryostat sections, non-neoplastic tissues removed from tumor samples and cells of interest manually dissected from the frozen block, with other tissues trimmed away [18]. Fresh-frozen prostate samples were provided by the Irish Prostate Cancer Research Consortium (PCRC) Bioresource, Conway Institute of Biomolecular and Biomedical Research, University College Dublin, Ireland; following approval from the Mater Misericordiae University Hospital ethics committee and written informed consent from all patients. The curated samples were selected based on the disease status of “aggressive” or “indolent”. Aggressive disease samples (n = 6) were selected from patients with biochemical recurrence (Median 27 months) and the median Gleason score was 7 with evidence of capsular invasion. Indolent cancer samples (n = 5) were selected from patients with no biochemical recurrence and had a median Gleason score of 5, with no evidence of capsular invasion. Matched non-malignant tissues (n = 11) were also obtained from this cohort. Tissue histology was further confirmed by an expert pathologist upon re-section of each tissue specimen and subsequent H&E staining. Based on the histology findings, a portion of prostate tissue, containing the highest tumor content, was cut from the specimen. These sub-samples were stored at −80°C before RNA extraction.

### Quantitative RT-PCR

Quantitative RT-PCR was performed on patient samples from the Irish PCRC cohort. RNA was extracted from non-malignant (n = 11), indolent (n = 5) and aggressive cancer tissues (n = 6). Reverse transcription of 500 ng of total RNA was performed using a high-capacity cDNA Reverse Transcription Kit (Life Technologies Pty. Ltd., Cat# 4368814). Real-time PCR was performed using 2 μL cDNA for each of the following TaqMan^®^ gene expression assays (Single Tube TaqMan^®^ Gene Expression Assays Life Technologies); *APPL1* (Hs00179382_m1), *RAB5A* (Hs00991290_m1), *EEA1* (Hs00929215_m1), *PDCD6IP* (Hs00994346_m1), *NOX4* (Hs00418356_m1), *SORT1* (Hs00361760_m1) and TaqMan^®^ Gene Expression Master Mix in a total volume of 10 μL. Three biological replicates were performed consisting of three technical replicates. Expression of each gene was normalized to an average of *ALAS1* (Hs00963534_m1) and *HPRT1* (Hs02800695_m1) reference genes and relative quantities calculated by ΔCt.

### Statistical analysis

A Kruskal-Wallis test with Dunn's multiple comparison was performed to evaluate differences in gene expression between multiple groups in the Tomlins cohort. Data from the Glinsky cohort was analyzed using *K*-means clustering by Cluster 3.0 [19] to determine high and low gene expression groups. These groups were evaluated using Kaplan-Meier survival curves with differences determined using the Log-Rank (Mantel-Cox) test. The construction of tri-gene signatures also used *K*-means clustering methodology to cluster the expression values of the three genes from each tissue, resulting in two clusters representing high or low expression for each of the three genes. One-way ANOVA was performed to evaluate expression differences between non-malignant, indolent and aggressive prostate cancer tissues from the PCRC bioresource. All statistical tests were performed using GraphPad Prism 6.05 (GraphPad Software Inc., California, USA). Significant results showed greater than 95% confidence (*P* < 0.05).
